# Elucidating Multimodal Imaging Patterns in Accelerated Brain Aging: Heterogeneity through a Discriminant Analysis Approach Using the UK Biobank Dataset

**DOI:** 10.3390/bioengineering11020124

**Published:** 2024-01-26

**Authors:** Lingyu Liu, Lan Lin, Shen Sun, Shuicai Wu

**Affiliations:** 1 Department of Biomedical Engineering, College of Chemistry and Life Science, Beijing University of Technology, Beijing 100124, China; lingyuliu98@sina.com (L.L.); sunshen@bjut.edu.cn (S.S.); wushuicai@bjut.edu.cn (S.W.); 2 Intelligent Physiological Measurement and Clinical Translation, Beijing International Base for Scientific and Technological Cooperation, Beijing University of Technology, Beijing 100124, China

**Keywords:** accelerated brain aging, advanced brain aging, subtypes, heterogeneity, structural MRI

## Abstract

Accelerated brain aging (ABA) intricately links with age-associated neurodegenerative and neuropsychiatric diseases, emphasizing the critical need for a nuanced exploration of heterogeneous ABA patterns. This investigation leveraged data from the UK Biobank (UKB) for a comprehensive analysis, utilizing structural magnetic resonance imaging (sMRI), diffusion magnetic resonance imaging (dMRI), and resting-state functional magnetic resonance imaging (rsfMRI) from 31,621 participants. Pre-processing employed tools from the FMRIB Software Library (FSL, version 5.0.10), FreeSurfer, DTIFIT, and MELODIC, seamlessly integrated into the UKB imaging processing pipeline. The Lasso algorithm was employed for brain-age prediction, utilizing derived phenotypes obtained from brain imaging data. Subpopulations of accelerated brain aging (ABA) and resilient brain aging (RBA) were delineated based on the error between actual age and predicted brain age. The ABA subgroup comprised 1949 subjects (experimental group), while the RBA subgroup comprised 3203 subjects (control group). Semi-supervised heterogeneity through discriminant analysis (HYDRA) refined and characterized the ABA subgroups based on distinctive neuroimaging features. HYDRA systematically stratified ABA subjects into three subtypes: SubGroup 2 exhibited extensive gray-matter atrophy, distinctive white-matter patterns, and unique connectivity features, displaying lower cognitive performance; SubGroup 3 demonstrated minimal atrophy, superior cognitive performance, and higher physical activity; and SubGroup 1 occupied an intermediate position. This investigation underscores pronounced structural and functional heterogeneity in ABA, revealing three subtypes and paving the way for personalized neuroprotective treatments for age-related neurological, neuropsychiatric, and neurodegenerative diseases.

## 1. Introduction

The brain aging process elicits intricate alterations in both the structure and function aspects of the brain. This phenomenon manifests in various forms of degeneration, encompassing cortical thinning [1], increased white-matter atrophy and lesions [2], and diminished functional connectivity [3]. Despite the profound impact of aging on the brain, current investigations into brain aging, specifically the categorization of brain aging subtypes based on neuroimaging features, are at an early stage of development [4,5]. Researchers are navigating the complexities of understanding the diverse patterns associated with brain aging. The complexity is further heightened by the interplay among genetic, lifestyle, and environmental factors, all of which contribute significantly to the observed diversity in the brain aging process [6,7,8]. This apparent heterogeneity intrinsic to brain aging emphasizes the imperative of studying brain-aging subtypes to unravel the underlying mechanisms and variations of brain aging within the aging population [9].

Traditional brain-aging research has categorized brain aging into distinct categories: resilient brain aging (RBA) [10,11,12], normal brain aging, and accelerated brain aging (ABA), occasionally referred to as advanced brain aging [13,14]. Such studies have revealed that RBA is associated with a greater resistance to the risk of neurodegenerative diseases like Alzheimer’s disease (AD). Conversely, ABA represents a paradigm wherein aging processes within the cerebral domain proceed at an accelerated pace, surpassing the expected rate corresponding to an individual’s chronological age. This acceleration is evidenced by the manifestation of cerebral characteristics that appear older than anticipated. Discernible disparities in the brain-aging biomarkers of ABA have been well-documented within the context of neuropsychiatric disorders. Conditions such as schizophrenia, post-traumatic stress disorder, anxiety disorders, and depression exhibit conspicuous deviations in some brain-aging biomarkers [15]. This observation underscores the intricate interplay between ABA and the underlying pathophysiological substrates of neuropsychiatric disorders. The implication arises that ABA processes may serve as potential contributors to the etiology and progression of such conditions. Expanding upon extensive databases of normative aging, the analysis of MRI data emerges as a pivotal avenue for scrutinizing ABA within the cerebral milieu. One such methodology, the Brain Age Gap Estimation (BrainAGE) method [16], leverages machine-learning techniques to discern individual-level variability in brain-aging dynamics. This involves the utilization of standard MRI sequences, wherein a prediction model, derived from a learning sample comprising neurologically healthy adults, is deployed to estimate the apparent biological age of a new individual’s brain. In this process, the disparity between the estimated biological age and the subject’s chronological age constitutes the brain-age “gap” (BAG), a metric quantifying the extent to which a given brain appears comparatively “older” or “younger” relative to the individual’s chronological age. The BrainAGE method thus provides a sophisticated means of assessing and quantifying the accelerated aging phenomenon within the brain, offering insights into the individualized dynamics of cerebral aging beyond chronological timelines. Studies have established positive correlations between increased BrainAGE and numerous diseases, including obstructive sleep apnea [17], schizophrenia [18], AD [19], major depressive disorder [20], chronic poststroke language impairment [21], and Parkinson’s disease [22]. Therefore, elucidating the heterogeneity of ABA is crucial for understanding the underlying pathophysiological processes in brain aging [23].

Within the realm of neurological disorders, the application of unsupervised clustering algorithms stands as a pervasive methodology for conducting ABA subtype analyses. In a seminal study by Wrigglesworth et al. [24], 167 individuals exhibiting ABA were identified from a cohort of 326 community-dwelling older adults based on their BrainAGE metrics. The investigators proceeded to employ latent class analysis (LCA) on the ABA subjects, incorporating a comprehensive array of cognitive, lifestyle, and health measures. The results of the LCA revealed the presence of two distinct ABA subtypes. The first subtype exhibited a low prevalence of obesity, a diminished likelihood of low general cognitive status, a smaller probability of low mental quality of life (QoL), and a reduced likelihood of low physical QoL. In contrast, the second subtype was characterized by a higher prevalence of hypertension, a lower probability of high general cognitive status, moderate scores in mental QoL, and a diminished likelihood of high physical QoL. These findings underscore the utility of unsupervised clustering in unraveling nuanced health-related subtypes within the context of ABA. Unlike unsupervised learning, semi-supervised learning methods utilize labeled and unlabeled data to train a base classifier to distinguish between target and control groups, which is then updated in an unsupervised manner to discover the heterogeneity of the target group. This approach leads to more accurate predictions and a deeper understanding of the disease. Eavani et al.’s study [25] in ABA, utilizing the Mixture of Experts (MOEs) method on MRI data from 400 participants aged 50 to 96, identifies 5 distinct ABA phenotypes. This research underscores the importance of capturing the heterogeneity and subtypes of ABA rather than seeking a single signature, providing insights for future studies in understanding the neurobiological underpinnings of ABA. However, this study confronts two notable challenges. First, the limited dataset, encompassing approximately 261 subjects displaying ABA that were derived from around 400 participants, may raise concerns about the generalizability of the results. Additionally, while the MOE framework amalgamates the unsupervised modeling of mixtures of distributions with the supervised learning of classifiers, bestowing it with commendable merits in subgroup identification and multivariate pattern discrimination, it is not without its shortcomings. The MOE method’s integration of classification with clustering strategies leads it to inherit the limitations inherent in traditional clustering methods, particularly in the context of high-dimensional data where sparsity and dimensionality challenges prevail. With escalating dimensionality, the notion of distance between data points loses its meaning and becomes increasingly inadequate for discerning inherent patterns. This predicament is further exacerbated by the high sparsity endemic to high-dimensional spaces, yielding clustering outcomes that are marked by instability and unreliability. In this context, even minor perturbations in data points can yield entirely disparate cluster assignments, thereby compromising the robustness and consistency of the clustering results. In stark contrast, the recently developed heterogeneity through discriminant analysis (HYDRA) [26] was used for this study. A multiple max-margin discriminative analysis framework algorithm offers a promising and innovative solution. HYDRA’s prowess lies in its remarkable capacity to effectively capture neuroanatomical subtypes by utilizing multiple linear hyperplanes to create a convex polytope that distinctly separates various subgroups. Notably, HYDRA leverages the modeling capabilities of linear support vector machines (SVMs) to discriminate between homogeneous classes, even within high-dimensional data spaces. Moreover, HYDRA adopts a sophisticated two-pronged approach to improve upon its predecessor. Firstly, it meticulously initializes the iterative algorithm with great care, with a specific emphasis on promoting clustering solutions that exhibit diversity related to disease characteristics. This is achieved through the application of determinantal point processes (DPPs) to sample a wide array of aging directions, thus refining the initial clustering assignments. Secondly, HYDRA acknowledges the variability inherent in estimated solutions, particularly in non-convex settings, and skillfully employs a multi-initialization strategy in tandem with a fusion step. This comprehensive approach results in the production of robust and consistent results that accentuate the underlying group structure while simultaneously minimizing the impact of noisy perturbations. Overall, the innate advantages and advanced methodologies of HYDRA position it as a compelling and superior choice for heterogeneous analysis. Consequently, HYDRA has garnered widespread adoption in disease subtype analysis and recognition as the preeminent heterogeneous analysis algorithm in current practice [26,27,28,29,30].

In the course of this scientific investigation, BrainAGE functions as the pivotal tool for the stratification of the aging population into ABA and RBA subpopulations. Subsequent to this stratification, our study delves into the nuanced task of estimating diverse aging trajectories within the ABA population relative to the RBA. This intricate analysis is facilitated through the utilization of multimodal MRI image features. This research methodology signifies a departure from previous studies as it entails the examination of a notably expansive dataset comprising 5152 subjects. Within this dataset, 1949 subjects are representative of the ABA subpopulation, while 3203 represent the RBA subpopulation. The data were meticulously sourced from the UK Biobank (UKB) database, a reservoir of information that spans multiple distinct imaging modalities. Specifically, the brain imaging-derived phenotypes (IDPs) encompass 207 features derived from structural magnetic resonance imaging (sMRI), 144 features from diffusion magnetic resonance imaging (dMRI), and 210 features from resting-state functional magnetic resonance imaging (rsfMRI). This comprehensive approach substantially fortifies the robustness of our analysis, enabling a more nuanced understanding of the intricate interplay between ABA subpopulations and their corresponding neuroimaging profiles. By meticulously dissecting these multimodal MRI features, our investigation aims to contribute novel insights into the complex landscape of ABA, thus advancing our comprehension of the underlying mechanisms at play within the aging process.

In this study, we make three crucial contributions. Firstly, our investigation conducts a thorough examination of ABA subtypes through the application of multimodal neuroimaging techniques. To our knowledge, this study stands as the first-ever exploration into ABA heterogeneity utilizing multimodal neuroimaging on a significant scale, drawing from a substantial cohort of healthy volunteers (*n* = 31,621). This addresses challenges associated with limited sample sizes in previous research, ensuring a more comprehensive understanding of the diverse ABA population. Secondly, the high dimensionality (*n* = 561) poses challenges in heterogeneity analysis algorithms, prompting the introduction of the innovative HYDRA method for scrutinizing brain-aging heterogeneity. This approach effectively addresses inherent limitations in traditional methods applied to the analysis of ABA heterogeneity, showcasing its potential to unravel intricate patterns within high-dimensional neuroimaging datasets. This promises a fruitful avenue for future research into brain-aging heterogeneity. Beyond these advancements, the study’s contributions extend to the broader significance of understanding brain aging. Stratifying ABA subjects into three subtypes establishes a foundation for personalized prevention approaches against conditions like dementia. In essence, this study not only propels the methodological landscape of neuroimaging research forward but also holds profound implications for translational applications in the realms of personalized medicine and preventative neurology.

The organization of the remainder of this paper is as follows. In Section 2, a comprehensive introduction unfolds, elucidating essential facets such as the UKB data, the neuroimaging processing pipeline, the machine-learning model employed for brain-age prediction and the identification of ABA subgroups, and a meticulous overview of the statistical procedures that underpin this study. Section 3 meticulously unveils the study results, with a particular focus on the nuanced analysis of ABA subtypes. Following the presentation of results, Section 4 engages in a comprehensive discussion that contextualizes the findings within the broader landscape of neuroimaging research and the understanding of ABA, while a concise summary is encapsulated in Section 5.

## 2. Materials and Methods

### 2.1. Participants

The data utilized in this investigation emanated from a population-based prospective cohort study, namely the UKB [31], which is accessible at www.ukbiobank.ac.uk (accessed on 11 January 2021). The UKB had previously secured ethical approval from the North West Multi-centre Research Ethics Committee (REC reference 11/NW/0382). Furthermore, the research initiative documented herein had received approval from the UKB, designated by application number 68,382. During in-person interviews, a standardized questionnaire was employed to systematically acquire an extensive array of lifestyle information from the study participants. Additionally, the cognitive status of the subjects was evaluated using a touch-screen questionnaire. The UKB encompassed a comprehensive cohort, comprising a total of over 500,000 individuals.

As part of the overarching UKB study, a subset of participants underwent neuroimaging procedures, resulting in the acquisition of brain imaging data. To ensure data homogeneity, each of the three imaging centers was equipped with identical scanners and fixed platforms, maintaining consistency by refraining from major software or hardware updates throughout the study. Specifically, each center utilized a 3T Siemens Skyra (Skyra 3T, Siemens Healthcare GmbH, Erlangen, Germany) with software platform VD13 and a 32-channel receive head coil dedicated to brain imaging. The T1-weighted MRI employed a Magnetization-Prepared Rapid Gradient-Echo (MPRAGE) sequence characterized by a high spatial resolution of 1 × 1 × 1 mm, an image matrix of 208 × 256 × 256 mm^3^, and inversion time (TI)/repetition time (TR) of 880/2000 ms. DMRI data acquisition encompassed two b values (b = 1000, 2000 s/mm^2^) with a spatial resolution of 2 × 2 × 2 mm, covering a comprehensive set of 100 distinct directions. This protocol incorporated a multiband acceleration factor of 3. RsfMRI was executed with specific acquisition parameters, featuring a spatial resolution of 2.4 × 2.4 × 2.4 mm, a TR of 0.735 s, an echo time (TE) of 39 ms, and a multiband acceleration factor of 8. These standardized imaging protocols contribute to the reliability and consistency of the acquired data across the study cohort.

**Figure 1:**
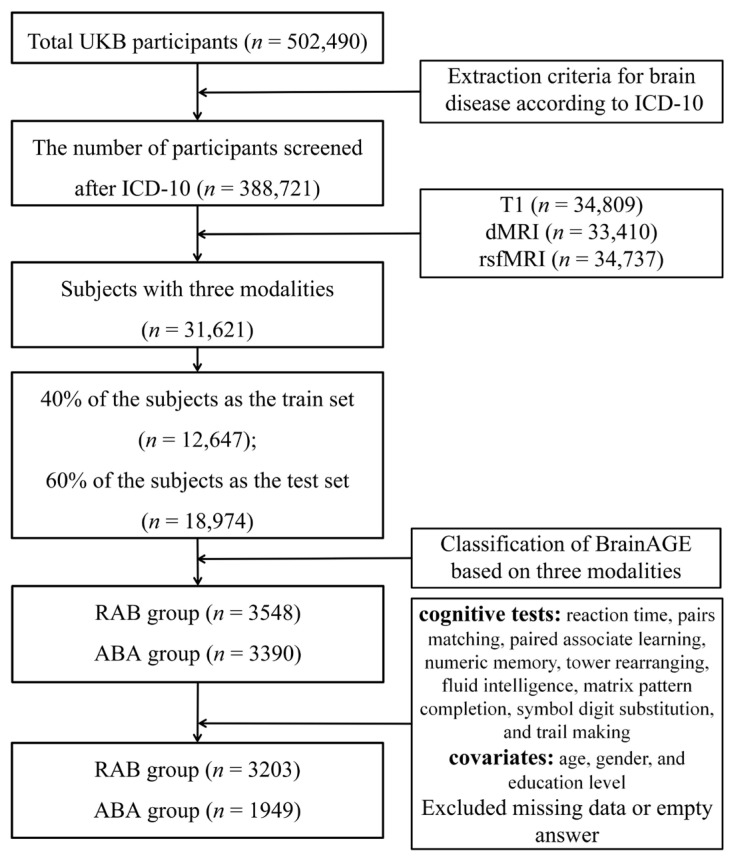
Flowchart depicting the subject screening process.

The selection process, detailed in the accompanying Figure 1, adhered to rigorous criteria aimed at ensuring data quality. Exclusion was based on the International Classification of Diseases, Tenth Revision (ICD-10), diagnostic classification system. Individuals diagnosed with malignant tumors of the eye, brain, and other parts of the central nervous system; cerebrovascular disease; psychiatric and behavioral disorders; neurological disorders; and other disorders affecting brain health were excluded from the analysis. This screening procedure led to the inclusion of 388,721 subjects between the ages of 45 and 83. Subsequently, 31,621 subjects possessing comprehensive sMRI, dMRI, and rsfMRI data were selected. This subset was then randomly divided into a training set (40%) and a test set (60%) to implement the brain-age prediction model. Within the test set, individuals exhibiting characteristics of ABA or RBA were identified using the brain-age prediction model. Specifically, individuals demonstrating a positive BrainAGE across all three imaging modalities were assigned to the ABA group. Conversely, those displaying consistently negative values in all three imaging modalities were assigned to the RBA group. Furthermore, participants who had not completed all nine cognitive tests, as well as those with incomplete data on covariates, were eliminated from the final analysis. As a result, the study ultimately comprised 1949 subjects classified within the RAB group, characterized by a mean age of 63.6 years with a standard deviation of 7.97. Concurrently, the ABA group encompassed 3203 subjects with a mean age of 64.6 years and a standard deviation of 6.96.

### 2.2. Imaging-Derived Phenotypes (IDPs)

The UKB presents a diverse array of neuroimaging modalities [32]. Following the meticulous acquisition of data, a standardized methodology is applied for image preprocessing and preliminary analysis, leading to a comprehensive set of IDPs. The carefully curated IDPs serve as the foundation for capturing valuable insights into different aspects of the brain structure and function, facilitating a comprehensive investigation aligned with the study’s objectives.

The T1 MRI, distinguished for its meticulous precision, stands as a structural modality acclaimed for its remarkable ability to intricately capture detailed brain anatomy at an impressive resolution. This imaging modality provides a potent contrast between gray and white matter, facilitating the accurate visualization of intricate brain structures. The quantification of volumes was meticulously conducted using the FMRIB software library (FSL, version 5.0.10), accessible at http://fsl.fmrib.ox.ac.uk/fsl (accessed on 16 February 2022). Employing the FMRIB’s automated segmentation tool (FAST, version FAST3), a total of 139 IDPs were derived. This was achieved by aggregating partial volume estimations within 139 regions of interest (ROIs) (UKB ID: 25782-25920) established in the MNI152 space, amalgamating parcellations from various atlases, including the Harvard–Oxford cortical and subcortical atlases (https://fsl.fmrib.ox.ac.uk/fsl/fslwiki/Atlases, accessed on 16 February 2022) and the Diedrichsen cerebellar atlas (http://www.diedrichsenlab.org/imaging/propatlas.htm, accessed on 16 February 2022). The warp field, previously estimated to effectuate the transformation of subject data into a standardized space, underwent inversion and subsequent application to the ROIs. This process generated a version of the ROIs in the native space, facilitating precise masking within the segmentation framework. Extraction of cortical thickness from cortical regions involved the meticulous implementation of the established FreeSurfer parcellation scheme [33]. This scheme, grounded in the Desikan–Killiany atlas, comprehensively delineates cortical domains across both hemispheres, encompassing a total of 68 discrete regions (UKB ID: 25755-26788, 26856-26889).

DMRI serves as a crucial tool for evaluating water molecule movement within the local tissue environment. At the voxel level, local estimates of diffusion properties provide valuable insights into microstructural tissue integrity, encompassing diffusion tensor estimates. Furthermore, long-range estimates derived from tractography, which involves the meticulous tracing of brain pathways, offer comprehensive information about the structural connectivity between pairs of brain regions. In this study, we employed the DTIFIT tool (available at https://fsl.fmrib.ox.ac.uk/fsl/fdt, accessed on 16 February 2022), to fit a diffusion tensor at each voxel. This procedure yielded multiple diffusion measures, encompassing fractional anisotropy (FA) and mean diffusivity (MD) maps. These collective measures provide a comprehensive elucidation of the characteristics of water diffusion within the brain tissue. Moreover, the dMRI data underwent sophisticated processing leveraging using NODDI (Neurite Orientation Dispersion and Density Imaging). NODDI enables the estimation of crucial white-matter microstructural parameter isotropic water volume fraction (ISOVF).

To delve into the intricacies of the white-matter microstructure, we employed tract-based spatial statistics (TBSS). TBSS facilitates the alignment of the FA image onto a standard-space white-matter skeleton through high-dimensional FNIRT-based warping. This standardized-space warp is subsequently applied to all other dMRI measures. Each resulting skeletonized image for dMRI measures underwent averaging across 48 standard spatial tract masks, meticulously defined by Susumi Mori’s group at Johns Hopkins University. This detailed averaging procedure produced a total of 144 distinctive IDPs (FA (UKB ID: 25056-25103), MDs (UKB ID: 25104-25151), and ISOVFs (UKB ID: 25440-25487)).

The analysis of rs-fMRI images was conducted using the MELODIC (Multivariate Exploratory Linear Decomposition into Independent Components) framework [34]. This processing pipeline integrated group principal component analysis and independent component analysis, culminating in the extraction of spatially orthogonal independent components (ICs) representing distinct resting-state neural networks. A low-dimensional group-independent component analysis approach was employed to obtain a population-level spatial map of the resting-state network. The functional images underwent pre-processing with 25 fractions (UKB ID: 25752), and a meticulous exclusion process eliminated 4 noise components, resulting in a set of 21 components of particular interest. Each of these components corresponded to unique resting-state networks, offering invaluable insights into the underlying neural activity patterns during rest. The online visualization of these ICs is facilitated through the Papaya viewer (https://www.fmrib.ox.ac.uk/ukbiobank/group_means/rfMRI_ICA_d25_good_nodes.html, accessed on 16 February 2022). This viewer, along with accompanying maps, provides an interactive and insightful platform for exploring and comprehending the spatial distribution of the ICs derived from rs-fMRI data. Moreover, a partial correlation matrix derived from rsfMRI data was utilized to represent the number of network connections, totaling 210 values. This was calculated by multiplying the 21 networks by 20 (excluding identity correlations) and dividing by 2, considering the matrix’s diagonal symmetry. The implementation of partial correlation aimed to enhance the precision of estimating direct “connections” between networks compared to full correlation.

### 2.3. Brain-Age Prediction Model

Lasso, short for “Least Absolute Shrinkage and Selection Operator,” is a statistical regularization technique in machine learning. It adds a penalty term to the regression equation, constraining the absolute size of the coefficients and effectively promoting sparsity by forcing some coefficients to be exactly zero. Lasso is widely employed in predictive modeling, particularly when dealing with high-dimensional datasets. Prior investigations into brain-age prediction [35,36] have consistently demonstrated the superior performance of the Lasso model when compared to other machine-learning models. Given these compelling findings, we have chosen the Lasso model as the method of choice for brain-age prediction in our study.

Within the Lasso model, the penalty regularization parameter, denoted as alpha, assumes a pivotal role in determining the intensity of the penalty applied to model parameters. The magnitude of alpha directly influences the strength of the penalties assigned to each parameter, resulting in varying degrees of model shrinkage. In the context of this study, we meticulously defined the grid search space for the alpha parameter as (0.001, 0.01, 0.1, 1, 10, 100). This specific range was chosen to efficiently explore the parameter space and identify the optimal alpha value that would maximize model performance.

BrainAGE [37] is a neuroimaging-based metric designed to quantify the difference between an individual’s actual chronological age and the predicted age of their brain. This innovative approach leverages structural brain imaging data to provide insights into the aging process at the neural level. The fundamental premise behind BrainAGE is to assess the extent to which the brain either accelerates or decelerates in comparison to the individual’s chronological age. BrainAGE also has a strong correlation with brain maintenance (BM) [38]. The brain-age prediction model entails the application of machine-learning techniques to brain imaging data, enabling the development of a predictive model for estimating the “age” of the brain through its imaging features. The BrainAGE score is derived by calculating the difference between the age predicted by the model and an individual’s chronological age (Equation (1)). A positive BrainAGE score suggests that the brain is aging at a faster rate than expected, potentially indicating accelerated aging or suboptimal BM. Conversely, a negative BrainAGE score implies a more youthful-appearing brain, indicative of better-preserved structural characteristics than what would be expected based on chronological age.

BrainAGE = Predicted age − Chronological age(1)

Recent studies have underscored the presence of a proportional bias in the computation of brain age, where the disparity between chronological age and predicted brain age exhibits a negative correlation with chronological age. This phenomenon is attributed to the well-documented effect of regression toward the mean [35,39]. This phenomenon has the potential to introduce bias in the prediction of age, which may be overestimated in younger subjects and underestimated in older subjects compared to their respective chronological ages. Given the inherent age-related bias, the imperative arises for the implementation of an age-bias correction procedure, as outlined in Equation (2).

Predicted age_corrected_ = Predicted age_raw_ − α − β × Chronological age (2)

where Predicted age_raw_ indicates brain age predicted by the Lasso model, and α and β represent the intercept and slope of the regression line between chronological age and predicted age in the training set.

Subsequently, subjects in the test sets were systematically categorized based on their BrainAGE. Individuals displaying positive BrainAGE values across all three modalities were categorized into the ABA group, indicating that their predicted brain age exceeded their chronological age. Conversely, subjects with negative BrainAGE values across the three imaging modalities were assigned to the RBA group, indicating a favorable condition where the predicted brain age suggested a structure and function younger than their actual age. This stratification provides a nuanced understanding of age-related deviations in brain structure and function, fostering a comprehensive characterization of individual differences in brain aging within the study cohort.

### 2.4. Non-Imaging Derived Phenotypes (Non-IDPs)

Throughout their active engagement in the UKB study, subjects were diligently queried to furnish comprehensive insights into their lifestyle and physical health using diverse methodologies. The amalgamation of this wealth of information culminated in the creation of non-imaging derived phenotypes (Non-IDPs), which serve as integral components of the broader analytical framework. The study comprehensively examined six Non-IDPs intricately associated with lifestyle and physical health. These variables included systolic blood pressure (UKB ID: 4080), time spent driving (UKB ID: 1090), hand grip strength (UKB ID: 46, 47), usual walking pace (UKB ID: 924), and diabetes diagnosed by a doctor (UKB ID: 2443).

### 2.5. Neuropsychological Tests

The neuropsychological battery, consisting of nine cognitive domains [40], served as the foundation for cognitive evaluation in this study. Specifically, two cognitive scales within the scope of our investigation—reaction time (UKB ID: 20023) and trail-making (UKB ID: 6350)—both incorporating time as a test outcome, underwent a log transformation to enhance their analytical robustness. A detailed overview of the neuropsychological tests is provided in Table 1.

### 2.6. Identification of ABA Subgroups Using HYDRA

Leveraging the information derived from IDPs, we employed the HYDRA algorithm to discern distinct ABA subtypes [26]. HYDRA is a semi-supervised machine-learning algorithm tailored for unraveling the intricacies of disease heterogeneity. In this study, this algorithm achieves ABA heterogeneity by partitioning ABA subjects, discerning patterns or transformations between subpopulations within the ABA group and a reference group (i.e., RBA subjects). The partitioning process employs a convex polytope, a construct amalgamating multiple linear max-margin classifiers. Notably, HYDRA demonstrates the capability to effectively regress out nuisance covariates, such as age and sex, enhancing its precision in discerning genuine patterns associated with brain aging. In its approach, HYDRA conceptualizes subjects as points within a high-dimensional space, aligning with the support vector machine (SVM) classification framework. Leveraging the discriminative power of linear SVMs in high-dimensional spaces, HYDRA extends this capability to the non-linear domain in a piecewise fashion. This extension involves the formation of a convex polytope through the combination of multiple hyperplanes, effectively segregating the two groups. Enclosed within this convex polytope are the RBA samples, while distinct faces of the polytope facilitate ABA subtyping. Each face encapsulates a distinct multivariate pattern of difference between the two groups, and hence a distinct accelerated aging process.

**Figure 2:**
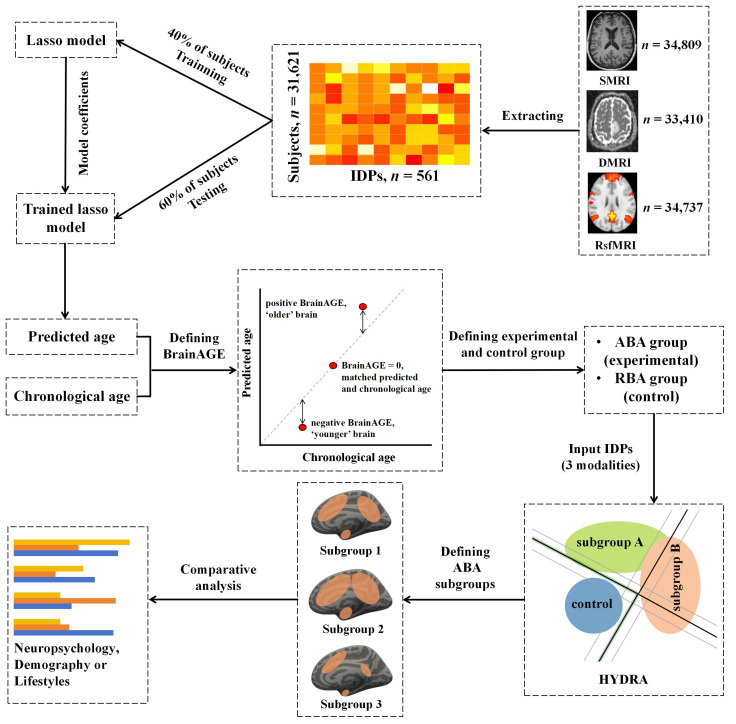
The comprehensive workflow of the present investigation.

In the initial phase, HYDRA allocates different labels to the ABA and control groups (RBA subjects). Subsequently, the algorithm integrates multiple linear max-margin classifiers into a convex polyhedron by clustering the k-values, where k represents the number of clusters, effectively distinguishing control subjects from those exhibiting ABA. The assignment of ABA subjects to the nearest hyperplane within a single linear subclassifier results in the division of all ABA subjects into K clusters, with each polyhedron encapsulating the distinct characteristics of an ABA subtype. The optimization problem is systematically addressed through an iterative procedure, alternately assigning ABA samples to the faces of the polytope and estimating hyperplanes to maximize the overall margin. This iterative coupling between clustering and classification serves the dual purpose of segregating ABA subjects based on accelerated brain-aging effects, rather than a global anatomical perspective. For optimizing the identification of ABA subtypes, a systematic approach was employed, ranging from two to five clusters, with five-fold cross-validation. Covariates, including age, gender, and education level, were considered during the process. Of note, the educational level underwent a transformation into years of education, aligning with established practices in prior research [41]. The stability of clustering outcomes was quantified using the adjusted rand index (ARI) [26] in conjunction with five-fold cross-validation. The determination of the optimal number of clusters relied on the maximum ARI, ensuring the selection of the most reliable clusters. The comprehensive workflow is depicted in Figure 2.

### 2.7. Statistical Analysis

The study encompassed three primary sections delineating distinct characteristics: (1) Lifestyle and determinants, encompassing variables such as age, gender, years of education, and six lifestyle factors pertaining to physical health; (2) Neuropsychological exam, comprising a comprehensive battery of nine cognitive assessments; and (3) IDPs derived from T1, dMRI, and rsfMRI, totaling 561 IDPs. For sections (1) and (2), differences between matched subtypes were rigorously compared. Disparities in qualitative variables were assessed using the chi-square test, while quantitative variables underwent analysis of variance (ANOVA). Two-by-two comparisons were executed utilizing Dunnett’s test, with a predefined statistical significance level set at *p* < 0.05. In section (3), the analytical framework encompassed a comparison of differences between subgroups and controls, employing ANOVA. To address the issue of multiple comparisons, the Bonferroni method was meticulously applied, imposing a stringent threshold of q < 0.01. All statistical analyses were conducted using SPSS 26 software, a widely acknowledged statistical package (SPSS, 1989; Apache Software Foundation, Chicago, IL, USA).

## 3. Results

### 3.1. Brain-Age Prediction

Within the scope of this investigation, Lasso regression analysis was selected as the preferred methodology for predicting brain age, with mean absolute error (MAE) serving as the metric for evaluating model performance. Interestingly, dMRI emerged as the modality with the highest predictive accuracy. The application of Lasso regression to dMRI data resulted in a remarkably low MAE of 4.03 years, indicating the effectiveness of this approach in estimating brain age. Moreover, the predictive accuracy based on T1 data, encompassing cortical thickness and gray-matter volume, resulted in an MAE of 4.17 years, while rsfMRI demonstrated an MAE of 5.28 years.

The categorization of ABA and RBA groups was contingent upon the consistency of positive or negative BrainAGE across the three modalities within the test set of brain-age prediction (*n* = 18,974). Specifically, if BrainAGE across all three modalities was positive, the subject was categorized as ABA; conversely, if BrainAGE was consistently negative, the subject was designated as RBA. This delineation led to the selection of 3203 subjects in the RBA group (mean age = 63.6 ± 7.97) and 1949 subjects in the ABA group (mean age = 64.6 ± 6.96).

### 3.2. Definition of ABA Subgroups

**Figure 3:**
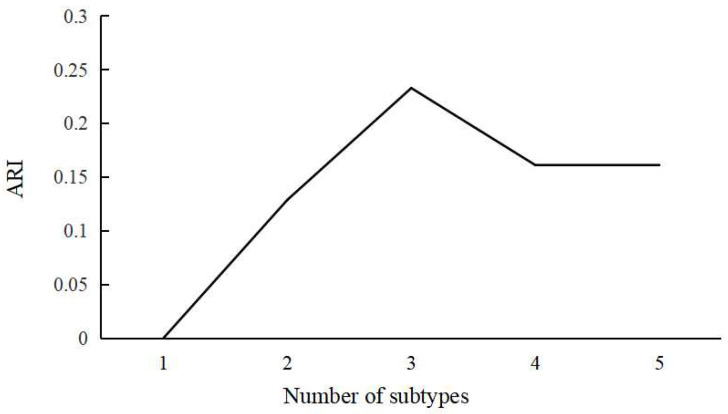
The ARI values correspond to varying numbers of subtypes.

Within the confines of this investigation, the HYDRA framework was implemented to partition ABA heterogeneity, where the ABA population assumed the role of the experimental group, and the RBA population served as the control group. Subsequent to this partitioning, meticulous scrutiny of the fidelity of cluster assignment transpired. The examination involved systematically varying the cluster number from 1 to 5, employing the ARI as the metric for assessment. The ARI quantifies the similarity between true and predicted cluster assignments, offering a measure of clustering accuracy that accounts for chance. Notably, a monotonically increasing trend was observed within the range of 1 to 3 subtypes. However, as the subtype count extended to 4 and 5, a relative decline in the ARI values was discerned in comparison to the trinary configuration. This observation suggests that clustering efficacy may be optimized into three distinct subtypes (refer to Figure 3). It is imperative to note that HYDRA employed a robust five-fold cross-validation strategy. The delineation of optimal subtypes reflects the outcomes observed in the validation sets across these folds. Subsequent analysis delineated that, based on the cross-validation result, 783 individuals from the ABA cohort were allocated to SubGroup 1, while SubGroup 2 comprised 561 ABA subjects, and SubGroup 3 encompassed 605 ABA subjects.

The comprehensive delineation of demographic information, as meticulously presented in Table 2, highlights the nuanced distinctions within these demographic variables. Substantial statistical distinctions in age and sex distribution were evident within the tripartite classification of ABA subjects. Notably, there were no discernible differences in years of education. To mitigate the potential confounding effects stemming from these demographic variations, the Generalized Linear Model (GLM) for IDps incorporated three crucial demographic variables—namely, age, sex, and years of education—as covariates. Through this inclusion, their respective influences were systematically controlled and eliminated. The application of rigorously controlled covariate regressions serves to enhance the precision of subsequent analyses and facilitates a nuanced interpretation of the influence of specific subtypes on the observed outcomes.

Following this, an ANOVA was employed to scrutinize discrepancies among RBA and distinct subgroups within the ABA cohort. In response to the inherent challenge of multiple comparisons, the Bonferroni method was applied with a stringent threshold (q < 0.01). Remarkably, this comprehensive examination revealed nuanced differences in the patterns of sMRI, dMRI, and rsfMRI features across the three subtypes. These findings underscore the intricate nature of neuroimaging alterations within distinct subtypes of ABA cohorts.

**Figure 4:**
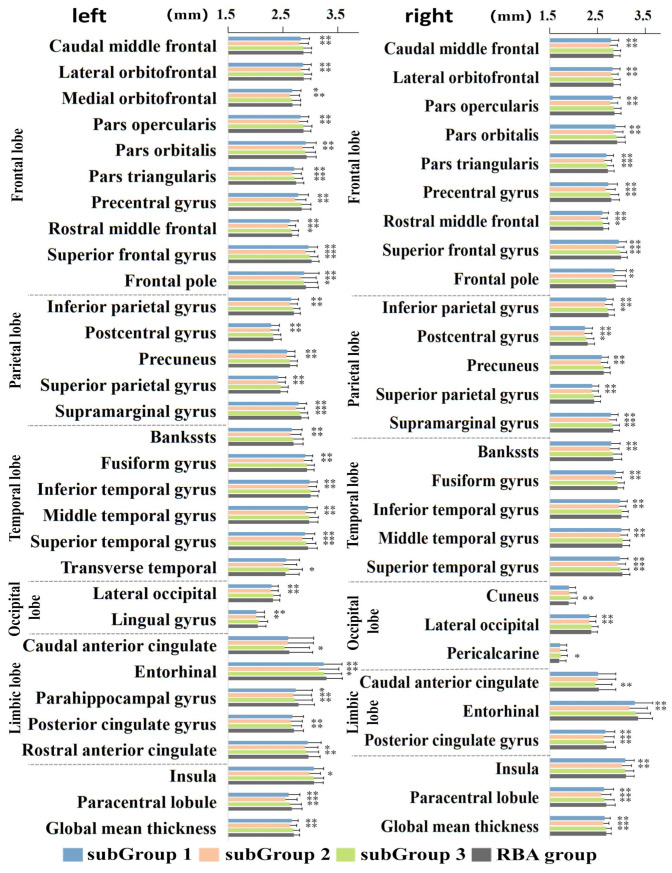
Comparative analysis of cortical thickness across the three ABA subgroups and the control group. Statistical significance denoted as * indicates q < 0.01, while ** signifies q < 0.001.

**Figure 5:**
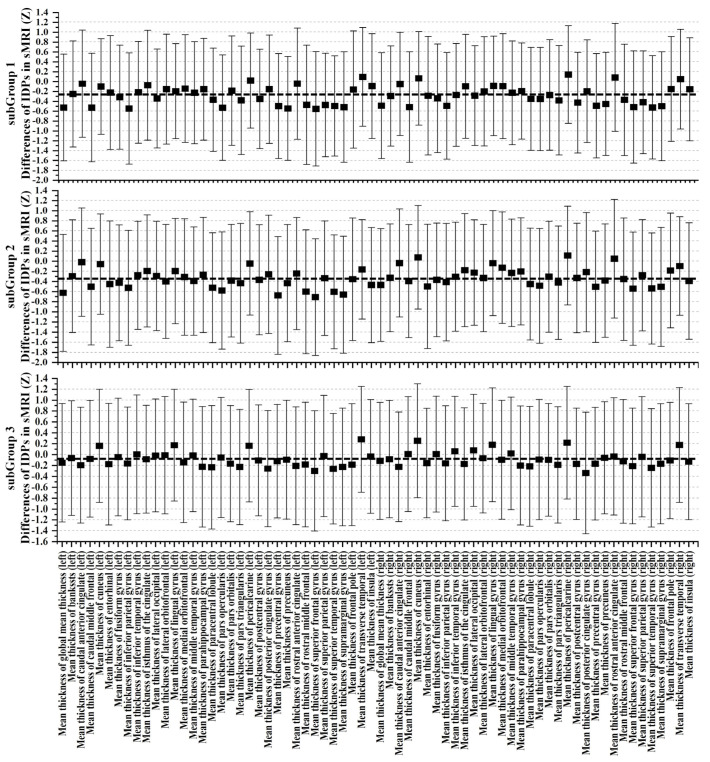
Examination of cortical thickness through Z-values for each thickness IDPs across the three ABA subgroups in comparison to the RBA group. Error bars represent the 95% confidence intervals. The black dotted line represents the mean Z-value for each subgroup.

In the context of structural alterations discerned through sMRI, Figure 4 and Figure S1 in Supplementary Materials have been meticulously crafted to provide comprehensive insights into the distinctions among the three identified ABA subgroups and the control group. SubGroup 1, consisting of 783 elderly subjects, exhibited diffuse cortical atrophy spanning the frontal, parietal, and temporal lobes bilaterally, with limited atrophy observed in the occipital and limbic lobes. This subgroup displayed extensive gray-matter volume reduction throughout the entire brain, emphasizing significant atrophy in key regions such as the Insula, Paracentral lobule, and Angular gyrus. SubGroup 2 demonstrated a comparable pattern of atrophy to SubGroup 1, with slight variations noted in the left cortex of the limbic lobe and Insula. In contrast, SubGroup 3 manifested small cortical and gray-matter volume atrophy, indicating regionally sparse and mild whole-brain atrophy. To further elucidate these morphological alterations, a graphical representation (Figure 5) of Z-values and their 95% confidence intervals for cortical thickness comparisons has been incorporated. This graphical representation unveils similar regions of atrophy across the three subgroups, yet discernible differences exist in the overall distribution pattern of atrophy. It is imperative to underscore that all Z-values were computed with respect to the mean and standard deviation of the RBA group, where Z-values for the RBA group serve as the baseline with a value of 0. SubGroup 3 exhibited the least pronounced atrophy, while SubGroup 2 showcased the most severe atrophy. These findings offer nuanced insights into structural distinctions among ABA subgroups, unraveling the intricacies of ABA-related morphological alterations.

**Figure 6:**
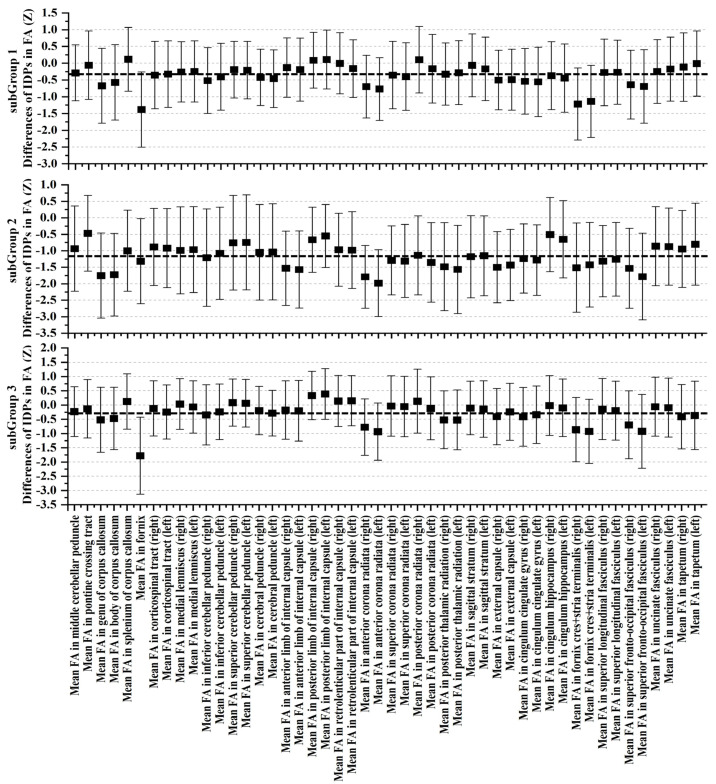
Examination of FA through Z-values for each FA IDPs within the three ABA subgroups in contrast to the RBA group. The error bars depict the 95% confidence intervals. The black dotted line represents the mean Z-value for each subgroup.

**Figure 7:**
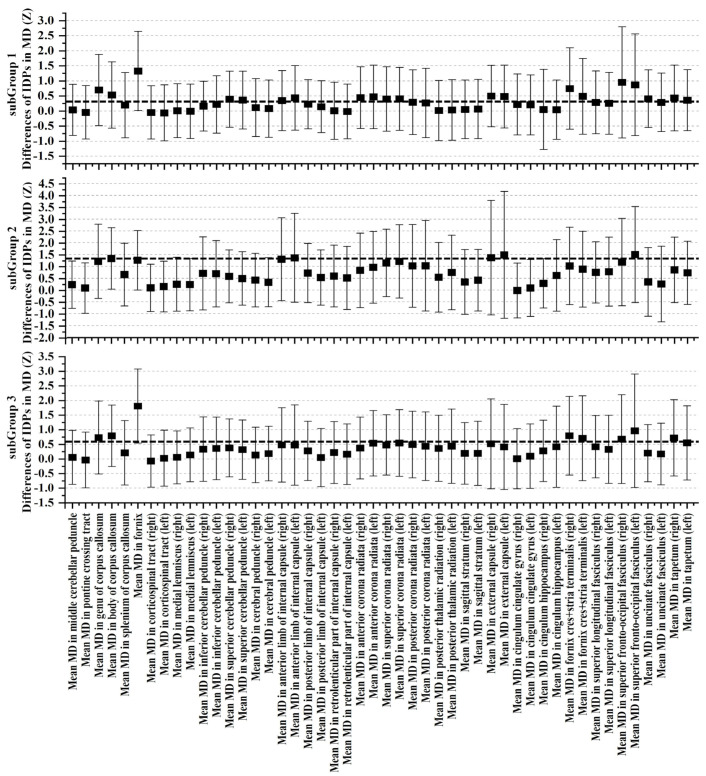
Examination of MD through Z-values for each MD IDPs within the three ABA subgroups in contrast to the RBA group. The error bars depict the 95% confidence intervals. The black dotted line represents the mean Z-value for each subgroup.

**Figure 8:**
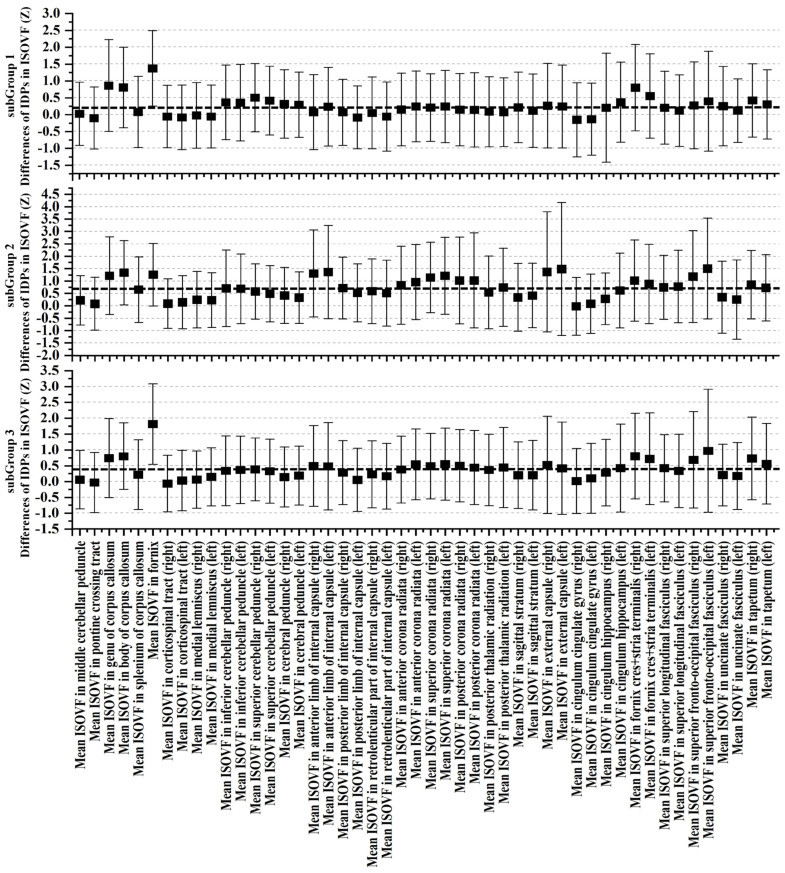
Examination of ISOVF through Z-values for each ISOVF IDPs within the three ABA subgroups in contrast to the RBA group. The error bars depict the 95% confidence intervals. The black dotted line represents the mean Z-value for each subgroup.

Upon meticulous examination of white-matter microstructure using dMRI, SubGroup 2 emerged as a focal point characterized by substantial deviations from the control group, indicating pronounced alterations across nearly all scrutinized regions. In comparison to the control group, SubGroup 1 and 3 also manifested a comprehensive array of distinctions from controls, albeit with a noticeably lower magnitude than observed in SubGroup 2. Graphical representations, as depicted in Figure 6, Figure 7 and Figure 8, unravel the nuanced variations in Z-values and their 95% confidence intervals for FA, MD, and ISOVF. All Z-values are computed relative to the mean and standard deviation of the RBA group, serving as the baseline with an expected value of 0. In the context of white-matter integrity, lower FA values and elevated MD and ISOVF values are indicative of compromised microstructural integrity. Remarkably, SubGroup 2 exhibited lower Z-values in FA compared to the other two subgroups, accompanied by higher values in MD and ISOVF. This observation underscores a pronounced degradation of white-matter integrity in SubGroup 2. In contrast, the Z-values within SubGroups 1 and 3 exhibited comparable trends, displaying closer proximity to 0. Contrary to the control group, SubGroup 1 displayed a compromised white-matter microstructure akin to that observed in SubGroup 3, albeit with a milder impact.

**Figure 9:**
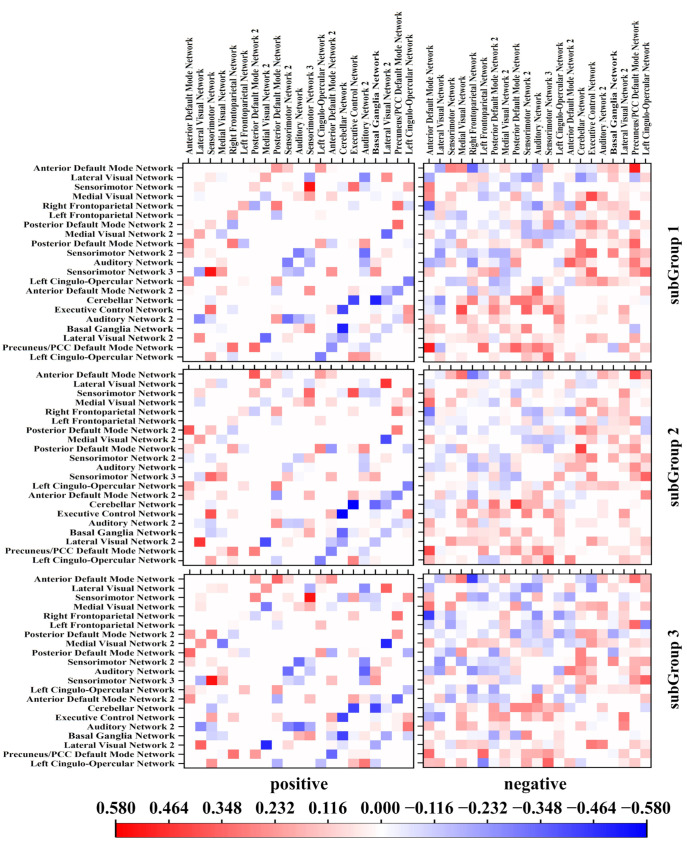
Heat map illustrating connection strength. The connection strengths within the three ABA groups were stratified based on the positive and negative phases of the RBA groups.

Examining functional connectivity through rsfMRI, this study meticulously delineates the intricate connection strengths between distinct ABA subgroups and the control cohort. The categorization of connection strengths within the control group, discerned through positive and negative connections, facilitated the comparison of Z-values for the three subgroups, elegantly presented as a heatmap in Figure 9. The Z-values presented in the analysis are derived in relation to the mean and standard deviation of the RBA group, establishing 0 as the baseline for Z-values in the RBA group. Noteworthy observations emerge as groups 1 and 3 exhibit a more analogous pattern in both positive and negative connection strengths. However, SubGroup 3 stands out with notably more negatively linking nodes within the negative connection category. In stark contrast, SubGroup 2 presents a divergent pattern characterized by a smaller change in negative connection strengths in comparison to the control group.

### 3.3. Cognitive and Non-IDPs Characteristics between Matched Subtypes

Detailed cognitive characteristics among the three ABA subtypes and the RBA are elucidated in Table 3. SubGroup 2 prominently exhibited the most discernible cognitive impairment, notably differing from the other subtypes in reaction time, symbol digit substitution, and trail-making. In contrast, SubGroup 3 displayed superior cognitive performance across all tests, demonstrating significant differences, particularly in fluid intelligence and matrix pattern completion, compared to the other subgroups.

**Figure 10:**
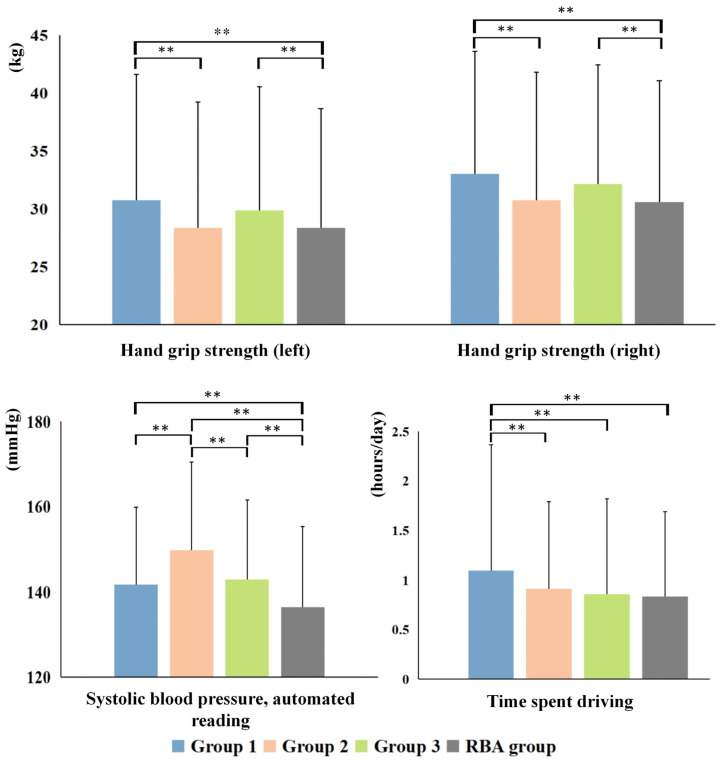
Quantitative analysis of Non-IDP variables among the three subtypes and the RBA group employed ANOVA, followed by pairwise comparisons using Dunnett’s test. Significance is denoted by ** at *p* < 0.001.

**Figure 11:**
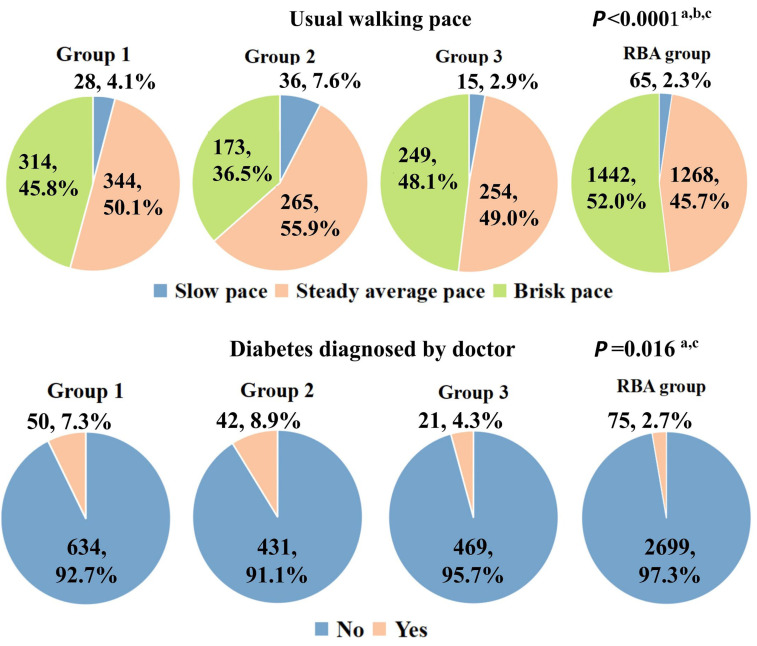
Comparative assessment of qualitative Non-IDP variables between the three subtypes and RBA group were then underwent by ANOVA, two-by-two comparisons were conducted employing Dunnett’s test. a: SubGroup 2 is significantly different from SubGroup 3 (*p* < 0.05); b: SubGroup 1 is significantly different from RBA group (*p* < 0.05); c: SubGroup 2 is significantly different from RBA group (*p* < 0.05).

Shifting the focus to Non-IDPs, differences among the subtypes are illustrated in Figure 10 and Figure 11. SubGroup 2 exhibited the most pronounced distinctions compared to the other two ABA groups, featuring elevated blood pressure, diminished grip strength, a higher prevalence of confirmed diabetes, and a slower pace in usual walking. SubGroups 1 and 3 displayed relatively fewer differences, primarily diverging in the time spent driving and usual walking pace. Concurrently, notable distinctions were observed in blood pressure, confirmed diabetes prevalence, and usual walking pace between SubGroup 2 and the RBA group. However, no statistically significant differences were identified in grip strength values and driving time. In the case of SubGroup 1, marked disparities were evident in all Non-IDP variables as compared to the RBA group, except for the prevalence of diagnosed diabetes.

## 4. Discussion

Harnessing the capabilities of HYDRA in conjunction with the distinctive datasets provided by the UKB study, our research endeavors sought to scrutinize the existence of neuroimaging-defined subtypes within a cross-sectional sample of ABA. Our analyses discerned the presence of three discernible subtypes, each characterized by distinct neuroimaging profiles. These three subtypes manifest distinctive attributes of brain gray-matter structure, white-matter microstructure, and functional network connectivity. Notably, SubGroup 3 displayed the mildest atrophy, resembling SubGroup 1 in white-matter microstructure and functional connectivity strength. In contrast, SubGroup 2 exhibited no significant atrophy disparities compared to SubGroup 1; however, SubGroup 2 is characterized by the most impaired white-matter microstructural integrity and displays distinctive connectivity networks. This differentiation implies potential variations in underlying aging mechanisms, shedding light on the intricate heterogeneity inherent in the aging process.

### 4.1. Complex Landscape of ABA

Within the broader spectrum, age-related cognitive impairment seldom emerges as a consequence of a singular disease entity. Instead, it presents as a multifaceted interplay involving diverse factors, encompassing AD, various forms of dementia, and a range of health conditions like traumatic brain injury, stroke, depression, or developmental disabilities. The escalating apprehension regarding age-related cognitive decline arises from its widely recognized role as a pivotal determinant shaping the overall quality of life [42]. Given this backdrop, there is a heightened emphasis on the pursuit of biomarkers capable of assessing individual brain age and forecasting the trajectory of cognitive decline.

Methodologies deployed to ascertain brain age, grounded in neuroimaging data, are designed to elucidate deviations in age-related cerebral changes. This is accomplished through the establishment of robust reference curves for RBA and ABA, providing personalized metrics of brain age. Importantly, these approaches are tailored to accommodate the multidimensional patterns that characterize the aging process within the brain. Such sophisticated strategies hold considerable promise for advancing our understanding of cognitive aging and facilitating proactive interventions to enhance cognitive well-being in the aging population.

In the course of this comprehensive investigation, the ABA cohorts were meticulously characterized based on the discerning metric of BrainAGE, as detailed in Table 4. Within both sMRI and dMRI modalities, SubGroup 2 consistently exhibits the highest BrainAGE levels, indicative of the most pronounced accelerated aging. However, in the realm of rsfMRI, SubGroup 2 demonstrates the lowest BrainAGE, portraying a distinctive profile of accelerated aging within this specific modality. Shifting the focus to the domain of dMRI-defined BrainAGE, SubGroups 1 and 3 demonstrate comparable BrainAGE levels, both of which are lower than that of SubGroup 2. Delving deeper into the analysis of rsfMRI-defined BrainAGE, SubGroup 1 emerges as the category with the highest values, yet it exhibits proximity to SubGroup 3.

Numerous determinants intricately shape and modulate the trajectories of individual brain aging. The application of neuroimaging-based models in exploring brain aging has yielded compelling insights. Notably, robust correlations have been unveiled between ABA, AD severity, and the prospective decline in cognitive functions [43]. Additionally, associations have been established between ABA and mild cognitive impairment (MCI) [44], as well as the conversion to AD [45]. Furthermore, investigations have linked ABA to diverse factors such as traumatic brain injury [46], HIV [47], chronic pain [48], and type 2 diabetes mellitus [49]. ABA has proven indicative not only of diminished physical and mental fitness but also of heightened allostatic load and increased mortality [50]. Moreover, individual brain aging exhibits noteworthy connections with an array of health parameters, personal lifestyle choices, and drug utilization [19]. Education levels and engagement in physical activity have also emerged as significant determinants influencing the ABA process [51]. This intricate interplay underscores the multifaceted nature of brain aging, weaving a complex tapestry of connections with various health indicators, lifestyle elements, and physiological conditions. The dissection of underlying mechanisms expediting brain aging not only enables researchers to identify intervention and prevention targets but also sheds light on the heightened risk of individuals experiencing ABA for conditions such as AD, Parkinson’s disease, and other neurodegenerative disorders.

### 4.2. ABA Subtype and Cognitive Reserve

In the realm of maintaining cognitive functioning amidst brain changes or insults, two pivotal forms of reserve come to the fore: brain reserve and cognitive reserve [52]. Brain age estimation serves as a valuable metric, providing a nuanced perspective on brain maintenance and reserves. Notably, ABA individuals, when compared to age-matched peers, exhibit compromised brain reserve capacities. This suggests that these individuals may face challenges in deploying alternative brain networks or cognitive strategies in the face of aging or insults. Cognitive reserve reflects the brain’s adaptive capacity against insults or aging [53,54]. Educational attainment, commonly employed as a proxy for cognitive reserve [55,56,57], reveals that ABA subjects, across three subgroups, possess educational durations exceeding 15 years, signifying a population with high cognitive reserve. The neural implementation of cognitive reserve manifests in two distinct forms: neural reserve and neural compensation [58,59]. Neural reserve posits variability in primary brain networks or cognitive paradigms underlying task performance, thereby offering resilience against brain aging. On the other hand, neural compensation describes the utilization of non-normally engaged brain structures or networks to compensate for aging-induced changes. These mechanisms exemplify the brain’s flexibility and adaptive strategies in the face of challenges. In the present study, the application of ICA facilitates the decomposition of fMRI data into distinctive brain networks. Positive connectivity within these networks signifies synchronized activity between networks, reflecting collaborative involvement in specific cognitive processes or tasks. The cooperative synergy inherent in positive connectivity is indispensable for the facilitation of streamlined information processing and the seamless execution of cognitive functions. Conversely, negative connectivity assumes a pivotal role in promoting cognitive flexibility, affording the brain the capacity to navigate between different cognitive states and alleviating interference among concurrent cognitive processes. Disparities in both positive and negative network connections observed between the ABA and RBA cohorts underscore a conspicuous neural compensation mechanism [60]. Specifically, the discernible augmentation in negative connectivity within ABA individuals suggests that, in the face of degeneration, the brain intensifies inhibitory interactions among disparate brain regions to counterbalance the disruptive effects of structural decline. In the specific context of ABA subtypes 1 and 2, despite structural similarities in neurodegeneration, nuanced differences in negative connectivity patterns are apparent. ABA subtype 1 prominently manifests a discernible proclivity towards cognitive compensation, indicating adaptive responses to the structural challenges inherent in neurodegeneration. Conversely, subtype 2 showcases a confluence of neural compensation and neural reserve. This observation underscores the inference that distinct strategies are employed by different subtypes within the ABA context, delineating nuanced approaches to addressing the intricacies of neurodegenerative processes.

### 4.3. Limitations

This study entails certain limitations that warrant careful consideration. Firstly, the exclusive utilization of data from the UKB introduces a notable limitation, as the subjects are predominantly of white ethnicity and hail from the United Kingdom. Consequently, the generalizability of the study findings to other countries or regions may be constrained. Secondly, in the implementation of HYDRA for semi-supervised learning, the RBA was deliberately chosen as the reference group. This decision stems from the discernible differences exhibited by the RBA when compared to the ABA cohort. Nevertheless, it is crucial to acknowledge that this choice may introduce potential bias into the subtype estimation. Thirdly, to validate the delineation of ABA subtypes, it is essential to broaden our experimental scope by integrating additional datasets and extending the spectrum of comparative analyses. However, a noteworthy limitation arises from the inherent inadequacies of outcomes derived from smaller datasets, which often lack the necessary representativeness. Moreover, the current state of the research landscape confronts a significant impediment characterized by a shortage of openly accessible datasets commensurate in magnitude to the UKB. This scarcity not only diminishes the depth of available data but also presents a formidable barrier to the facilitation of seamless cross-study comparisons. However, with increasing recognition from governments worldwide regarding the significance of large-scale neurobiological repositories in medical and clinical research [61,62], we anticipate a continual emergence of additional open-access large-scale biological databases.

## 5. Conclusions

Distinguishing itself from precedent investigations, this study capitalizes on considerable sample size and an extensive age spectrum, imparting significant robustness to the examination of brain variability within the ABA cohort. The utilization of the HYDRA methodology represents a notable methodological advancement, surpassing conventional heterogeneity analysis techniques used in ABA analysis. HYDRA not only discerns ABA subgroups but also enables the characterization of distinctions from the RBA group across multiple dimensions.

Looking ahead, the inclusion of subsequent follow-up waves from the UKB study promises a longitudinal exploration of the identified clusters. This longitudinal perspective is essential for unraveling the evolving nature of these clusters over time and elucidating their prognostic implications for brain and cognitive aging outcomes. The comprehensive insights derived from this study not only unveil inherent brain heterogeneity within ABA but also lay the groundwork for future analyses to deepen our understanding of the cognition and brain arising from the progressive ABA observed in UKB participants.

## Figures and Tables

**Table 1:** Cognitive domain, neuropsychological tests, and test descriptions.

Testing	Description	Cognitive Domain	UKB ID
Pairs matching	Number of incorrect matches made in round	Visual declarative memory	399
Numeric memory	Maximum number of digits remembered correctly	Working memory	4282
Fluid intelligence	Fluid intelligence score assessment	Verbal and numerical reasoning	20016
Paired associate learning	Number of correctly associated word pairs	Verbal declarative memory	20197
Matrix pattern completion	Number of correctly solved puzzles	Non-verbal reasoning	6373
Reaction time	Mean time taken to correctly identify matches	Processing speed	20023
Symbol digit substitution	Number of correct symbol digit matches made	Processing speed	23324
Tower rearranging	Number of correctly solved puzzles	Executive function	21004
Trail-making	Duration to complete alphanumeric path	Executive function	6350

**Table 2:** Demographics characteristics of RBA and ABA subgroups.

Characteristics	RBA Group	SubGroup 1	SubGroup 2	SubGroup 3	*p*-Values
*n*	3203	783	561	605	
Age (years)	64.62	61.57	66.78	63.40	<0.0001 ^a,b,c^
Education (years)	16.17	15.46	15.54	15.75	0.527
Women, *n* (%)	1884 (58.8%)	326 (41.6%)	259 (46.2%)	302 (49.9%)	0.008 ^c^

a: SubGroup 1 is significantly different from the SubGroup 2; b: SubGroup 2 is significantly different from the SubGroup 3; c: SubGroup 1 is significantly different from the SubGroup 3.

**Table 3:** Cognitive characteristics within the identified study subtypes.

Cognitive Function Test	UKB ID	RBA Group	SubGroup 1	SubGroup 2	SubGroup 3	*p*-Values
Pairs matching	399	3.577	3.664	3.814	3.540	0.307
Numeric memory	4282	6.819	6.554	6.452	6.688	0.089
Fluid intelligence	20016	6.820	6.307	6.435	6.927	<0.001 ^b,c^
Paired associate learning	20197	7.234	6.670	6.445	6.854	0.097
Matrix pattern completion	6373	8.227	7.756	7.745	8.088	0.036 ^b,c^
Reaction time	20023	2.764	2.764	2.784	2.769	<0.001 ^a,c^
Symbol digit substitution	23324	19.634	18.654	17.633	18.832	0.003 ^a,c^
Tower rearranging	21004	10.041	9.807	9.580	9.958	0.246
Trail-making	6350	2.711	2.733	2.765	2.718	<0.001 ^a,c^

a: SubGroup 1 is significantly different from SubGroup 2 (*p* < 0.05); b: SubGroup 1 is significantly different from SubGroup 3 (*p* < 0.05); c: SubGroup 2 is significantly different from SubGroup 3 (*p* < 0.05).

**Table 4:** BrainAGE of the ABA subtypes.

Group	sMRI	rsfMRI	dMRI
SubGroup 1	6.55 ± 4.51	11.51 ± 8.07	6.19 ± 4.55
SubGroup 2	7.85 ± 5.94	10.73 ± 7.79	7.19 ± 5.52
SubGroup 3	5.59 ± 4.39	11.41 ± 8.51	6.40 ± 4.64
RBA group	−6.19 ± 4.56	−10.85 ± 8.14	−5.91 ± 4.06

## Data Availability

The imaging datasets generated by the UK Biobank and analyzed in the present study can be accessed through the UK Biobank data access process, as outlined at http://www.ukbiobank.ac.uk/register-apply/ (accessed on 11 January 2021). The UK Biobank’s Research Access Administration Team impartially manages all data access requests from both academic and commercial researchers, without exhibiting any preference or exclusivity. Requests are diligently evaluated to ascertain their alignment with public health research interests, and if deemed supportive, are promptly approved. Comprehensive information regarding the available data from the UK Biobank can be obtained at http://www.ukbiobank.ac.uk (accessed on 11 January 2021). It is important to note that the precise count of participants with imaging data accessible in the UK Biobank may marginally differ from the figures delineated in this manuscript.

## Supplementary Materials

The following supporting information can be downloaded at: https://www.mdpi.com/article/10.3390/bioengineering11020124/s1, Figure S1: Comparative analysis of gray matter volume across the three subgroups and the control group.
