# Supplementary material for: Elucidating Multimodal Imaging Patterns in Accelerated Brain Aging: Heterogeneity through a Discriminant Analysis Approach Using the UK Biobank Dataset
Source: Bioengineering (Basel). 2024 Jan 26;11(2):124. doi: 10.3390/bioengineering11020124 (PMC10886122; doi:10.3390/bioengineering11020124)
Supplement: Supplementary file 1 [file bioengineering-11-00124-s001.zip › bioengineering-2800700-supplementary.pdf]

Figure S1. Comparative analysis of gray matter volume across the three subgroups and the control group. Statistical significance denoted as * indicates q< 0.01, while ** signifies q < 0.001.

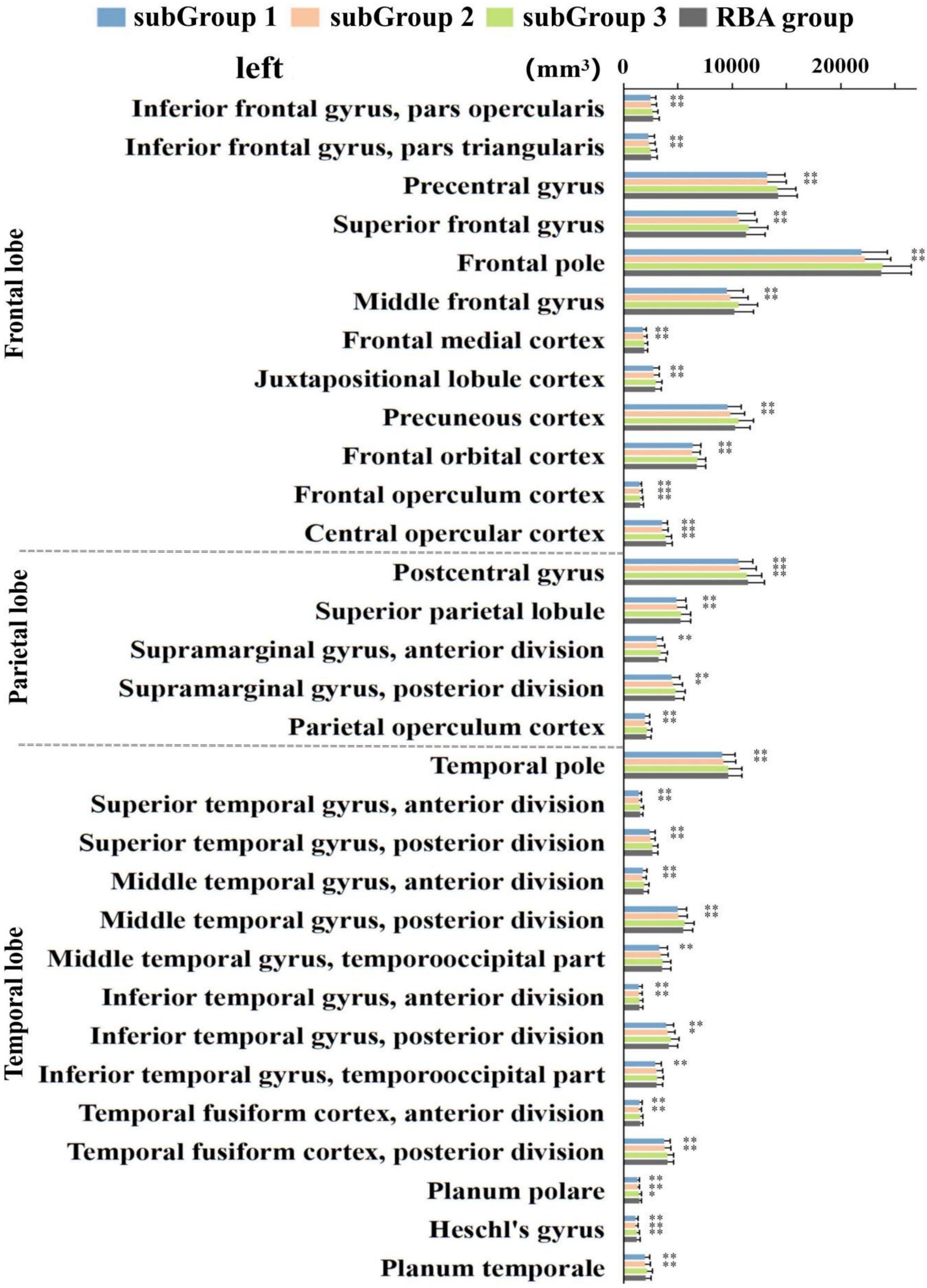

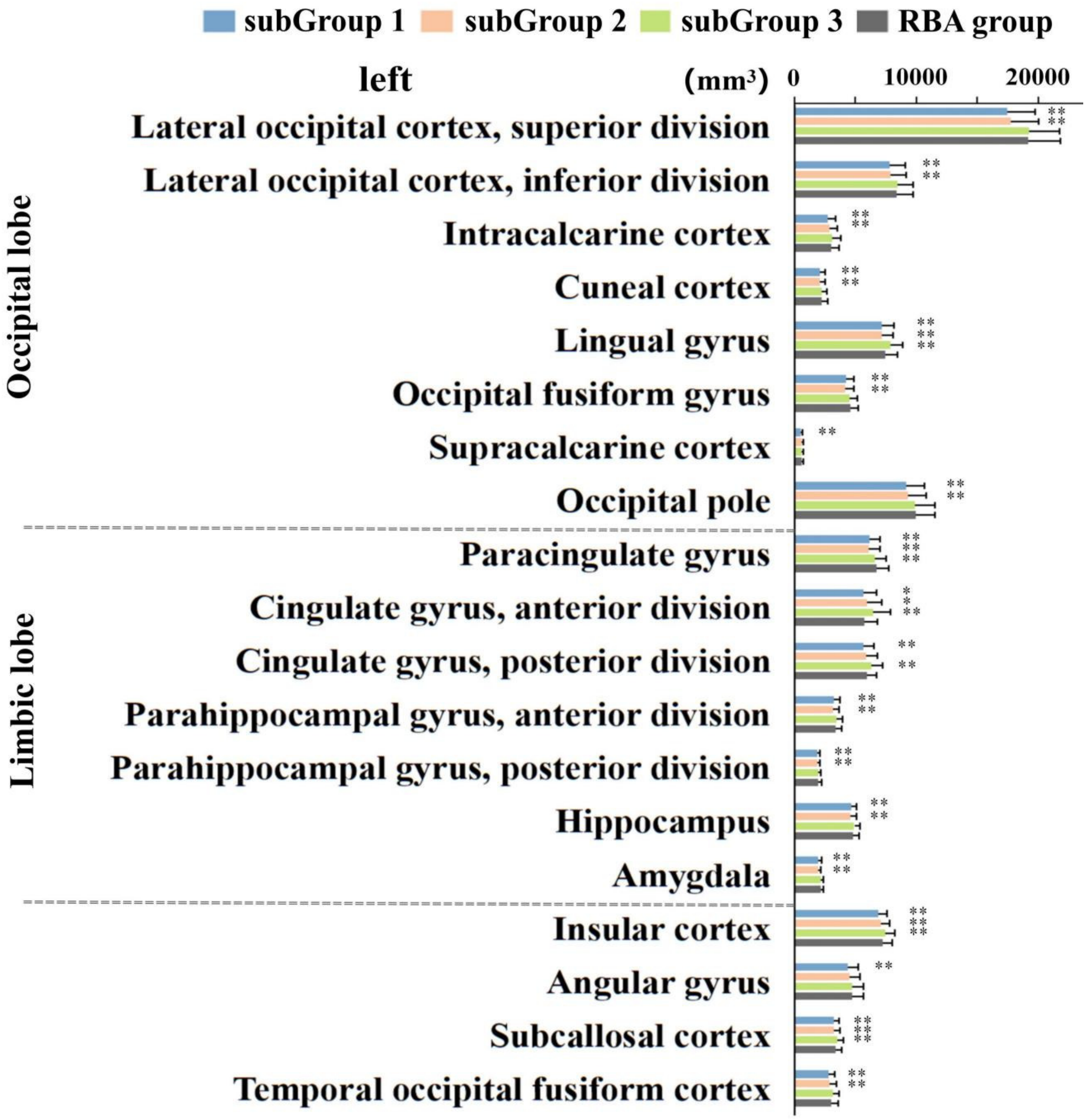
subGroup 1
subGroup 2
subGroup 3
RBA group
left
(mm³)
0
10000
20000
Occipital lobe
Lateral occipital cortex, superior division
Lateral occipital cortex, inferior division
Intracalcarine cortex
Cuneal cortex
Lingual gyrus
Occipital fusiform gyrus
Supracalcarine cortex
Occipital pole
Limbic lobe
Paracingulate gyrus
Cingulate gyrus, anterior division
Cingulate gyrus, posterior division
Parahippocampal gyrus, anterior division
Parahippocampal gyrus, posterior division
Hippocampus
Amygdala
Insular cortex
Angular gyrus
Subcallosal cortex
Temporal occipital fusiform cortex

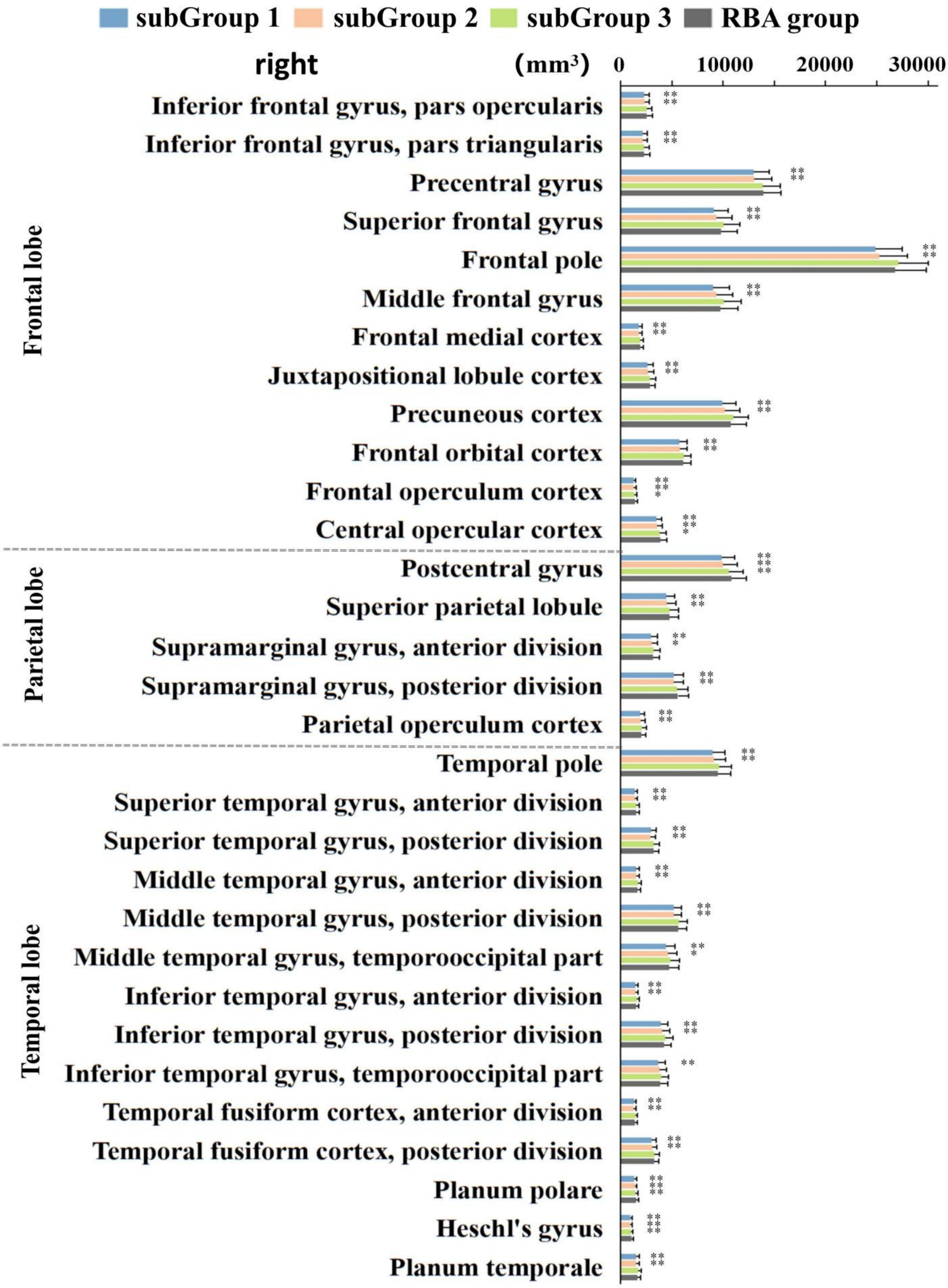

subGroup 1
subGroup 2
subGroup 3
RBA group
right
(mm³)
0
10000
20000
30000
Frontal lobe
Inferior frontal gyrus, pars opercularis
Inferior frontal gyrus, pars triangularis
Precentral gyrus
Superior frontal gyrus
Frontal pole
Middle frontal gyrus
Frontal medial cortex
Juxtapositional lobule cortex
Precuneous cortex
Frontal orbital cortex
Frontal operculum cortex
Central opercular cortex
Parietal lobe
Postcentral gyrus
Superior parietal lobule
Supramarginal gyrus, anterior division
Supramarginal gyrus, posterior division
Parietal operculum cortex
Temporal lobe
Temporal pole
Superior temporal gyrus, anterior division
Superior temporal gyrus, posterior division
Middle temporal gyrus, anterior division
Middle temporal gyrus, posterior division
Middle temporal gyrus, temporooccipital part
Inferior temporal gyrus, anterior division
Inferior temporal gyrus, posterior division
Inferior temporal gyrus, temporooccipital part
Temporal fusiform cortex, anterior division
Temporal fusiform cortex, posterior division
Planum polare
Heschl's gyrus
Planum temporale

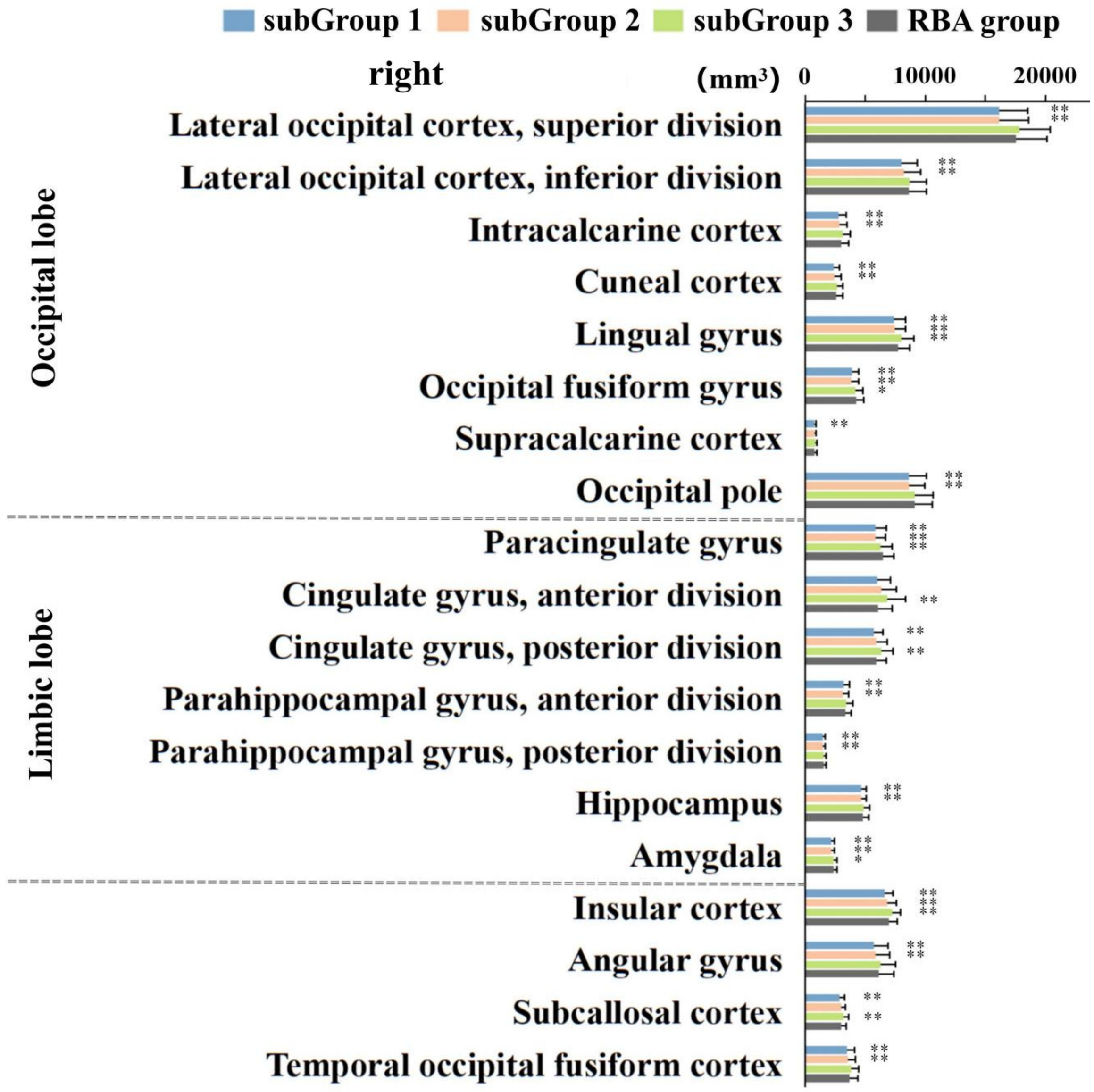
subGroup 1
subGroup 2
subGroup 3
RBA group
right
(mm³)
0
10000
20000
Occipital lobe
Lateral occipital cortex, superior division
Lateral occipital cortex, inferior division
Intracalcarine cortex
Cuneal cortex
Lingual gyrus
Occipital fusiform gyrus
Supracalcarine cortex
Occipital pole
Limbic lobe
Paracingulate gyrus
Cingulate gyrus, anterior division
Cingulate gyrus, posterior division
Parahippocampal gyrus, anterior division
Parahippocampal gyrus, posterior division
Hippocampus
Amygdala
Insular cortex
Angular gyrus
Subcallosal cortex
Temporal occipital fusiform cortex
